# The Biddenden Maids

**Published:** 1874-03

**Authors:** 


THE BIDDENDEN MAIDS.—We are indebted to Dr. Walter F. Atlee for a cake or cracker, about four inches long by two inches broad, which, with the circular that is copied below, was given him some years since by a patient who had lately come to this country from Biddenden, England. The man stated that the crackers were doled out year after year as directed by the will of the sisters, and as had been done regularly since their death. The circumstance is very interesting, as showing the way in which these old foundations are maintained in England for many centuries. Scientifically, the chief interest attaches to the fact that these two individuals, although so closely joined, lived for over thirty years. The union appears to have been more close than that between the Siamese Twins.

The cake is apparently a water-cracker, and is in the shape of a tomb-stone with a figure of the twins upon its face. This effigy represents two women joined together at the hips and shoulders, clad in short, very low, tight-laced bodices, and hooped skirts, with gigantic mammæ, of singularly perfect workmanship, above the bodices.

We have afforded this matter so much room in to-day's issue because, so far as we know, these ladies have not elsewhere figured in a scientific journal. As they were evidently covetous of posthumous fame, we doubt not that their manes will be most grateful to us for thus honoring them, after nearly eight centuries, during which they have waited for embalming.

We are informed that there is in existence a very old English book containing receipts and accounts of what are vulgarly known as "curiosities," in which these faithful sisters and fast friends are spoken of in detail. Unfortunately, we have not been able to obtain a sight of the volume.

We see no reason for doubting the authenticity of the record The statement that the twins died within six hours of each other is strong internal evidence of its truth, since an ignorant person making up an account would not be likely to conform so closely to the facts of experience.

